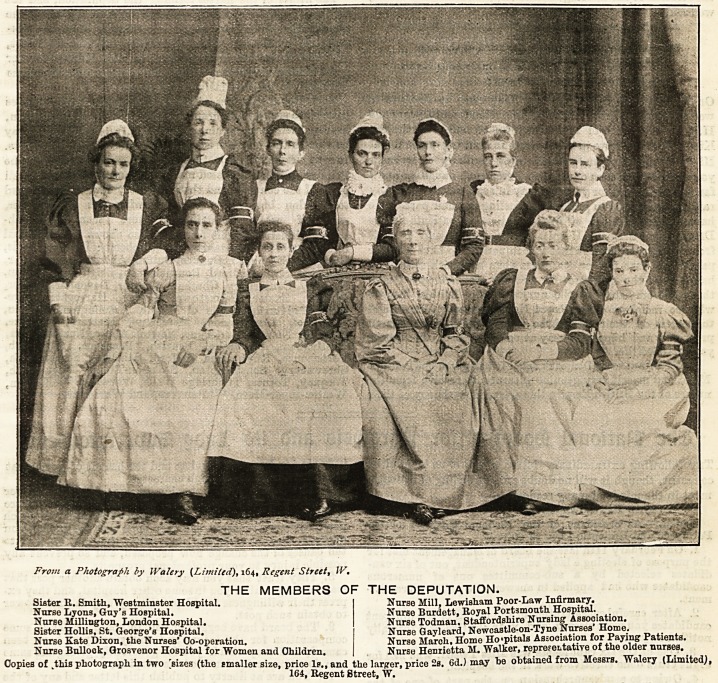# The Hospital Nursing Supplement

**Published:** 1896-03-21

**Authors:** 


"The Hospital" Nursing Mirror.

Being the Extra Nursing Supplement of "The Hospital" Newspaper.

[Contributions for this Supplement should be addressed to the Editor, The Hospital, 428, Strand, London, W.C., and should have the word "Nursing" plainly written in left-hand top corner of the envelope.]

### News from the Nursing World.

### ROYALTIES AMONG THE HOSPITALS.

That the Duchess of York's interest in hospitals is unabated has been abundantly shown lately, for several visits have been paid by Her Royal Highness and the Duke of York to the wards of the metropolitan hospitals. Their Royal Highnesses went to Guy's Hospital last week, and were conducted round the wards by Mr. E. H. Lushington (treasurer), Miss Nott-Bower (matron), and members of the staff.—The Princess of Wales paid a flying visit to Brighton the other day, but found time to go over the Alexandra Hospital for Children, nevertheless, in company with the Princesses Victoria and Maud.—The visit of the Duke and Duchess of York to Lancaster to open the new infirmary has been fixed for March 24th. They are timed to arrive at Lancaster at twelve o'clock, accompanied by the Lord and Lady Derby, and will be presented with an address of welcome.

### ROYAL HOSPITAL, RICHMOND.

The new "Princess May" Ward for children at the Richmond Royal Hospital which will be opened, it is hoped, some time during the coming summer, will be a very important addition to that institution, and one of which the want has been much felt. The new ward will contain eighteen beds. A spurt will have to be made by the friends of the hospital and by people in Richmond generally to raise the increased funds necessitated through the extension of its beds, and no doubt the money required will speedily be forthcoming. The Duke and Duchess of Teck and the Duchess of York take a warm interest in the well-being of the Richmond Hospital, which is shared by the Duke of Cambridge, who is its president.

### HAMPSHIRE NURSES' INSTITUTE.

The Hampshire Nurses' Institute (Southampton) has increased its staff from fourteen to nineteen, and it was stated at the recent annual meeting that the institute had done very much better since the number of nurses had been thus raised. The new year was begun with a balance in hand of £45, and the subscription list having been well kept up through the kindness of the friends of the institute, it has been possible to offer a higher rate of salary, and so secure the services of thoroughly competent nurses. The resignation of Nurse Little, after twenty-seven years on the staff, has been received with much regret, and the committee, in according to her a cordial vote of thanks for her long-continued faithful service, announced their intention of presenting her with a "monetary gift." Nurse Little has worked on the staff of the institute since its foundation.

### THE MALE NURSE IN AMERICA.

Glowing accounts have appeared in certain American papers of the future career opening before trained male nurses, of the high salaries commanded by these men, and the popularity of the profession generally, "more than four hundred applicants for positions in the New York Training School for Male Nurses being turned away last year." But some remarks on this subject in an American medical contemporary put a rather different complexion on the matter. Concurring with a statement that at the present time New York does not possess more than a dozen really good and competent male nurses, the writer goes on to say that "Some might go even farther and say there is no such thing as a good trained male nurse, but this is, perhaps, somewhat too radical. The fact is, however, that men do not make good trained nurses, and they are merely tolerated as necessary evils by those members of the profession who recommend their employment. There are certain classes of cases which men alone can properly manage, and for this reason there must be trained male nurses. The profession is, therefore, a legitimate and useful one, but the range of usefulness is not very wide and the field is not a great one. The qualities which make a man a good trained nurse are such as would usually make him a more successful worker in a pleasanter field. Therefore the most desirable men rarely continue long in this profession; indeed, many of them use it simply as a stepping-stone to graduating in medicine later. While male nurses do get large wages, their employment is not certain, they often have long periods of inactivity, and this irregularity of employment tends to irregularities and intemperance in living."

### MACCLESFIELD GENERAL INFIRMARY.

Miss Clara Bowman is leaving the Macclesfield General Infirmary, at which institution she has held the post of matron, at the end of April, on her approaching marriage. At the last monthly meeting of governors, when Miss Bowman's letter of resignation was read, the Chairman, Mr. Lockitt, J.P., proposed the following resolution: "That the resignation of the matron be accepted with sincere regret, and the governors wish to convey to Miss Bowman their hearty congratulations and best wishes for her future happiness, assuring her of their entire satisfaction with the conscientious and efficient manner in which she has discharged the responsible duties of matron, maintaining a high tone of discipline and good feeling throughout the institution." Many cordial expressions of goodwill came from the governors present, one and all testifying to the pleasant relations which had existed between the matron and everyone connected with the infirmary in a manner which could not fail to be very gratifying to Miss Bowman herself. We cordially join in congratulations and good wishes.

### ANOTHER WORKHOUSE "ACCIDENT."

How much longer, it may be wondered, will it be possible for such a horrible tragedy to occur as that just reported from the Wolverhampton Workhouse? Two pauper wardsmen, whether or not by the authority of the nurse nominally in charge of the wards is scarcely clear from the accounts to hand, attempted to bath an unfortunate paralytic inmate of the workhouse, Lovatt by name, who, on being put into the water, "screamed with pain . . . and afterwards alleged that he had been put into boiling water." At any rate, he subsequently died from "shock and inflammatory action following upon the scalds," which extended "from the loins to the toes." At the inquest a verdict of "Accidental death" was returned, and the jury added a recommendation that "no patient should be bathed without an order from a nurse, and that the nursing staff should be increased." The nursing staff apparently consisted of "one trained nurse," who was held responsible by the guardians for "six wards and sixty-six cases." It is the same old story of economy *versus* common humanity which comes from one workhouse after another time and again, and the sacrifice of a life or two and the infliction of any amount of suffering, by arousing public indignation, seems to be the only way of bringing home to some "guardians" (?) of the poor the cruelty of leaving sick and helpless paupers to the tender mercies of their fellow-inmates. It is no less cruel to the nurse, for no conscientious woman can stand the strain of such a charge, and we know at the present moment of more than one case where a workhouse nurse's health and prospects have been entirely sacrificed by the present system. When is the State going to exercise its proper authority and once and for all prohibit so-called "pauper nursing"? Why should a man be scalded to death because he is ill, poor, and helpless, and in the wards of a State institution?

### LIVERPOOL EYE AND EAR HOSPITAL.

A very successful three days' bazaar has been held at Liverpool, at the Hardman Street Assembly Rooms, in aid of the Eye and Ear Hospital, so seriously damaged by fire a few weeks ago. The bazaar was arranged as an Oriental street, the general effect being very attractive, though the lady stall-holders, in neat nursing uniforms of dark blue merino and pale blue print, with regulation caps, collars, and cuffs, looked a little out of keeping with their Eastern surroundings. "St. Paul's Stall" was in charge of the matron and some of the nurses from the hospital. It is hoped that a substantial sum may be handed over to the institution as a result.

### MIDDLESBROUGH NURSING ASSOCIATION.

Some confusion appears to exist in the lay mind with regard to the essential difference between private nursing and district nursing associations. This has lately been emphasised in the case of the Middlesbrough Nursing Association, of which a statement was said to have been made with reference to the distribution of the local Hospital Saturday Fund, that "there was an important difference between the nurses' home and the other medical charities, in that the former could make money by the services of the nurses, whereas the contributions to the hospital were purely voluntary." The Middlesbrough Association has two distinct branches, financially entirely separate—one for private nurses, which is self-supporting, and one for district nurses, which is a charity, and depends for its sinews of war upon the contributions of the charitable, and should in no way be confounded with the private nursing institution. As the hon. secretary of the Middlesbrough Association points out in a letter to the local press, in the event of the private nursing branch showing a profit on its working—which has not so far been the case—that profit could not in justice be used for any other purpose than for the immediate benefit of its nurses. The services of the district nurses are given freely and entirely gratuitously to the poor of Middlesbrough, and no institutions deserve more generously at the hands of the public than those which have for their object the relief of the poor in their own homes in times of sickness.

### PRIVATE NURSING ABROAD.

Inquiries reach us almost every week as to possible openings for private nurses in foreign countries; many correspondents apparently wishing to start on their own account in other lands. The advice invariably given, advice which is borne out by practical experience, is that unless nurses have very certain prospects of work awaiting them on their arrival in a fresh field of labour, or unless they have sufficient funds in hand to be able to afford a time of waiting, it is an act of folly to make the attempt. Someone recently asked a question with regard to private nursing prospects in Cairo, and to those who may be contemplating taking up work there, the following sentence from the letter of a Cairo correspondent may act as a timely warning. "Concerning private nursing in Cairo," writes an experienced nurse, "there is no opening unless engaged beforehand by a doctor. I have seen and helped many nurses there who were unable to pay their way or return home." The same remark might be made of many other places, and nurses are earnestly warned against leaving England for other countries with the vague idea of "finding work," or on mere chance assurances that employment is to be had for the asking.

### LECTURES AT CATERHAM.

Classes of instruction in anatomy and physiology have been inaugurated at Caterham Asylum, lectures on these subjects being given to the nurses by G. Campbell, Esq., assistant medical officer. Systematic instruction in theoretical and practical nursing is given by Mrs. Warren, the matron, and efforts are being made to raise the standard of asylum nursing in every possible way.

### SHORT ITEMS.

The nurses of the Fir Vale Infirmary, Sheffield, have just given their annual entertainment to the convalescent patients and inmates of the workhouse. An excellent programme was carried through, and much enjoyed by all.—A successful and much-appreciated course of lectures on nursing has been given at Appleby by Miss Musgrove. The attendance at each lecture averaged between 80 and 100 people.—The annual report of Villa Sunny-Bank Private Nursing Home, at Cannes, has just been issued. Several improvements have been carried out during the past year, and the value of such an institution, with all the comforts of skilled nursing which it offers, has been abundantly proved. The committee are anxious to purchase the freehold of the property, but increased funds are required to accomplish this desirable end.

### The Nurses' Wedding Present to H.R.H. Princess Maud.

By command of H.R.H. the Princess of Wales a deputation of twelve nurses attended at Marlborough House on Thursday, the 12th inst., to present the wedding present to Princess Maud, a drawing of which was published in THE HOSPITAL of February 29th, page cxci. The deputation was introduced by Mr. Burdett, and in addition to H.R.H. the President of the Royal National Pension Fund for Nurses, the Princesses Victoria and Maud and Prince Charles of Denmark were present. As the royal party entered the saloon at Marlborough House they were preceded by a delightful little Chinese dog, recently brought from Pekin by Sir Arthur O'Connor, which had a magnificent head and tail, and strutted in true drum-major fashion. The Princess of Wales having spoken to each of the nurses, Mr. Burdett made the presentation on behalf of the subscribers.

Mr. BURDETT said: I have the honour, by command of H.R.H. the Princess of Wales, to whom the nurses of the British Empire feel the deepest gratitude and loyalty, to offer for the acceptance of H.R.H. Princess Maud a Surprise Tea-table, which has been selected after much consideration as the wedding present of the nurses of the Royal National Pension Fund. The subscribers to this present reside in all parts of the United Kingdom, including places so far apart as Aberdeen and the Isle of Wight, Great Yarmouth and Aberystwyth, Plymouth and Dundee, Edinburgh and London, Portsmouth and Glasgow. Nearly 1,200 nurses spontaneously subscribed 1s. each to this gift, which represents an amount of loyalty and devotion to our Princess and President, and to the members of her family, which it would be difficult to adequately express in words. It may be interesting to state that among the many suggestions which were received as to the form this present should take, one nurse, with great moderation, suggested a silver teapot; whilst another, whose views were much wider, thought nothing less than a gold dinner service would be adequate to the occasion. Out of the abundance of the heart the mouth speaketh, and this may account for the proposal of another nurse who was in favour of a medicine chest. One post brought a letter urging that a silver bicycle should be presented to Princess Maud, but the writer sent a second letter by a subsequent post in which she stated she hoped her suggestion might not be regarded as improper, because she

*From a Photograph by Walery (Limited), 164, Regent Street, W.*

THE MEMBERS OF THE DEPUTATION.

Sister R. Smith, Westminster Hospital.
Nurse Lyons, Guy's Hospital.
Nurse Millington, London Hospital.
Sister Hollis, St. George's Hospital.
Nurse Kate Dixon, the Nurses' Co-operation.
Nurse Bullock, Grosvenor Hospital for Women and Children.
Nurse Mill, Lewisham Poor-Law Infirmary.
Nurse Burdett, Royal Portsmouth Hospital.
Nurse Todman, Staffordshire Nursing Association.
Nurse Gayleard, Newcastle-on-Tyne Nurses' Home.
Nurse March, Home Hospitals Association for Paying Patients.
Nurse Henrietta M. Walker, representative of the older nurses.

Copies of this photograph in two sizes (the smaller size, price 1s., and the larger, price 2s. 6d.) may be obtained from Messrs. Walery (Limited), 164, Regent Street, W.

had since heard a rumour that H.R.H. the Prince of Wales objected strongly to bicycles, and only permitted tricycles to be used at Sandringham. Ultimately it was determined to offer for Her Royal Highness's acceptance the Surprise Tea-table which I have now the honour to present on behalf of the nurses, because it was felt that it might be frequently used by Her Royal Highness, and that, whenever it was used, it might bring to her mind the pleasant remembrance of the thousands of nurses working in all parts of the British Empire who have the warmest and tenderest feelings of sympathy for Princess Maud. I may, perhaps, be permitted to remind Prince Charles that Mr. William Forsyth, 33 years ago, in a poem which he wrote on the occasion of the marriage of H.R.H. the Princess of Wales, well said—

And the queen of our hearts is the queen of the sea,
And as long as the sea rolls on
May the love of the faithful, the faith of the free
Be around her children's throne.
. . . . For who but the sea-king's child should be
The bride of the Brave Old Land?

On the present occasion the Sea King came to England to woo and win the bride of the brave old land, as 33 years ago H.R.H. the Prince of Wales had wooed and won the Sea King's child. I have great pleasure in assuring your Royal Highness that the nurses desire most cordially to welcome you as the bridegroom of our dear President's daughter, and I fancy their regard for yourself may in some measure, at any rate, be attributed to professional feeling on their part. We have a great number of Danish sailors as patients at the Dreadnought Seamen's Hospital at Greenwich, and I know from my own experience that it is impossible to have a better patient to care for or to nurse than a Danish sailor. I have now the pleasure to present to Princess Maud this table on behalf of the nurses of the British Empire who are members of the Royal National Pension Fund, and to express on their behalf the most earnest wish that all prosperity and happiness may attend you both throughout your married life.

Prince Charles of Denmark said: It has given the Princess Maud and myself very genuine pleasure to receive from the nurses of the British Empire this splendid testimony of their good wishes and kindly feelings towards us both. I can assure you, and through you all those who have contributed to this present, that we shall very frequently use, and never cease to value this tea-table, which will ever remind us of the volume of kindly feeling and sympathy extended towards us by so many nurses, for whose calling and work we have the profoundest admiration.

Each nurse was then presented to their Royal Highnesses, and the Princess of Wales, the Princesses Victoria and Maud, and Prince Charles spoke to and shook hands with each member of the deputation. The Princess of Wales invited the nurses to tea, which they thoroughly enjoyed and greatly appreciated.

The names of the nurses forming the deputation, and the institutions they represent, are to be found at the foot of the photograph, which was taken immediately after the presentation by Messrs. Walery (Limited), 164, Regent Street, W. The nurses are most grateful to Miss Knollys for her great kindness in looking after their comfort and happiness during their visit to Marlborough House.

Their Royal Highnesses Princess Maud and Prince Charles have since sent a photograph to each nurse of the deputation, bearing their autographic signatures. It is needless to say how much this gracious act is appreciated by the recipients. It was very noticeable that the list of subscribers to the Princess' present consisted mainly of nurses residing in all parts of the country, and that relatively few were attached to London hospitals. The following list of localities, each of which contributed more than one subscriber, illustrates this fact, and also shows the universal interest which was taken in the scheme: London, Paris, Glasgow, Edinburgh, Dundee, Dublin, Aberdeen, Axminster, Barnsley, Bath, Bedford, Bournemouth, Brighton, Brixham, Bromley, Canterbury, Chatham, Chester, Devonport, Dover, Durham, Exeter, Felixstowe, Halifax, Harrogate, Hereford, Huddersfield, Isle of Wight, Leamington, Lincoln, Liverpool, Llandrindod, Maidstone, Malvern, Margate, Matlock, Newcastle-on-Tyne, Newton Abbot, Northampton, Nottingham, Pembroke, Penrith, Penzance, Plymouth, Portsmouth, Rhyl, Ross, Salford, Salisbury, Scarborough, Shrewsbury, Southampton, Stoke-on-Trent, Teignmouth, Torquay, Totnes, Tunbridge Wells, Warwick, Wellington, Weston-super-Mare, Whitehaven, and Worcester.

### The National Hospital for Paralysis and its Lady Superintendent.

The following extraordinary letter seems to us to need no comment, though it will no doubt create a feeling of astonishment in the minds of many readers:—

To Miss Fynes Clinton.

Madam,—As president of the National Hospital for the Paralysed and Epileptic I beg to state—

1. On February 11th last the board of management met for the purpose of electing a lady superintendent, out of six candidates selected by a sub-committee out of numerous candidates who had applied in answer to a public advertisement.

2. After careful consideration of the merits of the different candidates you were elected, and your election was formally notified to you.

3. One of the six candidates was Miss Tweed, who had for eight years done effective work in the hospital.

4. Owing to a misapprehension on the part of one of the members of the board as to Miss Tweed's claims to election by reason of her past services, and to a mistake whereby her testimonials were not submitted to the board, her claims were ignored.

5. At a subsequent meeting of the board specially called on February 26th to reconsider the case, it was decided to request you to extricate the board from the difficult position in which they had placed themselves by resigning.

6. The board wish to state that your resignation was asked for not because they felt any sort of doubt as to your qualifications, but because they felt that they had not done full justice to Miss Tweed's claims. The board feel great regret in having to lose your services and the benefit of your great experience, ability, and high character.

7. The board hereby tender to you their thanks for acquiescing in their request, and offer to you their sincere apologies and regrets for the injustice and injury to which you have been subjected, and they hope that the explanation here given will remove any impression that may exist as to the causes of the change now proposed, and so prevent any possible injury to your professional prospects.

8. They trust that you may soon obtain another post that may be congenial to you in some other hospital, and they express their willingness to aid you in any way in their power to obtain such post.

9. The board have begged of you to accept £100 as some compensation for the injury and annoyance you have been caused, and they thank you for generously giving the same as a donation to the funds of the hospital.

10. You are at liberty to publish this letter and any of the previous correspondence that has passed between you and any member of the board, the secretary, or solicitor, and to make use of the same in any way you may think fit.

I am, yours faithfully,
(Signed) Westminster.

Eaton, March 10th, 1896.

### Wants and Workers.

Will any readers of The Hospital help a nurse by orders for crochet petticoats or plain needlework? Apply to Mrs. Field, 135, Vauxhall Bridge Road, Westminster.

### Training School Registries.

A paper read at the Superintendents' Convention, Philadelphia, Feb. 12th, 1896, by Louise Darche, Superintendent New York City and City Hospital Male Training Schools, Blackwell's Island, New York.

### II.—Registry and Club.

The Graduates' Co-operative Registry is still in the future, but I hope in the near future. The first step towards such a registry should be a graduates' club, chartered and legally authorised. This club might be composed of graduates of one school or of several schools.

We will suppose a club thus formed in some large city with a membership of one or two hundred, and in good working order. What would be easier than for such a club to appoint a committee of trusted members to consider the question of a registry in connection with club management? When the report was ready and a set of rules drafted a mass meeting of club members could be called, and the whole question submitted and voted upon. Or, better still, some popular and well-trusted member could be proposed as agent for a term of from three to six years at a fixed salary, with the understanding that this appointment should take effect only when an assured membership for the registry would guarantee sufficient financial reforms.

The popular wish for the agent having been ascertained, and rules and regulations formulated, the next step of finding out the proportion of club members who would wish to benefit by and support the registry could be easily ascertained by the pledge system. If sufficient returns were received by a set time the doctors and general public could then be notified on what day and where the registry would be opened, and the registry year could start from that day.

I have suggested appointing the agent for a set term of years, as only thus could an enterprising woman be secured for the office, and only thus could permanency (one of its radical requisites) be secured in the registry management. In the rules it would be well to have this term definitely fixed, also the amount of registry membership fee to be paid, the percentage on earnings if any, and the schedule of rates nurses will charge for their services, &c.

The club-room could be used as the registry office, and hus save considerable expense. A committee, probably the original committee, could be appointed on registry management. This committee should meet with and report to the club managers at stated meetings, and the club managers should be considered ultimately responsible for the enterprise when once it is started. Should the percentage system be adopted, and more funds accumulate than were necessary for the registry expenses, the club should have the option of using this fund for enlarging or improving its quarters, providing a club library, or for any other legitimate purpose in connection with the club or registry. It might be well, however, as a wise precautionary measure, to have a considerable floating capital in the bank in case the registry membership should fluctuate, or a year of unusual health in the community should result in a dearth of nursing work, and of consequent low returns in percentage to the registry.

It is most important that the physicians or other patrons of the registry should understand from the first that good nurses can always be obtained by writing, telephoning, or calling at the registry. It is not necessary that applicants for nurses should see or talk with the nurse providing the agent can guarantee a good one, though sometimes they will prefer to do so. They should never be obliged to look up the nurse herself at her lodgings. It should distinctly be the agent's duty to see that the nurse who is asked for, or to whom the call is sent, should be notified, and should report herself to the doctor or at the patient's house at the specified hour. A doctor may have but a few minutes in his day to devote to the business in securing a nurse for a case, and if by stepping to his telephone, or by making a hasty call at a registry office, he can secure the kind of nurse he wants and at the hour he wants her, he will surely feel grateful to that agency and bound to patronise it.

Many nurses object to having a price fixed for their services. They say they should do as the doctors do, charge according to the fortune or income of the patient's family, or the difficulty of the case. This is a fallacy. Nurses are not doctors, nor do they work under the same conditions as doctors do. Doctors do not get their cases through a directory or registry. A doctor can afford to charge little or nothing at one case because at the same time he may have another case well able to pay him double. A nurse can have but one case at a time. Neither, as a rule, is a nurse engaged at first hand as a doctor is by a family, she usually comes as the doctor's assistant, chosen by him, and he, in a sense, feels responsible both for her and her charges.

There should be a definite fixed charge known from the start, and when a patron of the registry asks the inevitable question, "How much do your nurses charge?" the agent should be in a position to give a definite answer according to a schedule of prices with which she should be provided.

Probably the best schedule of rates is that fixed on the graded basis. A certain set of nurses who have been doing private nursing for some time successfully may not wish to accept less than 25 dols. per week. A second set find that in the long run they will do better by offering to nurse for 21 dols. per week. A third set, lately graduated perhaps, and not yet "in demand," may be willing to register for 16 dols. or 18 dols. per week. By this system the agent would be able to meet a fair question with a fair answer, and to supply nurses according to the ability of the applicant to pay for them.

Before leaving this subject, there is one more point we should consider, and that is, the bearing or effect the protective registry has upon training school management. A wise woman who contemplates entering a training school course will naturally consider what prospects will lie before her after she has gained her diploma. The school which has fostered a registry, and by this means established a footing which the newly-graduated nurse may enter upon as soon as she finished her hospital work, will naturally be looked upon with more favour, and will secure a better class of applicant than the one which leaves its graduates to shift for themselves and secure recognition and work as best they may.

But some will say that this is a protective system, which applies only to the graduates in large cities. What is to become of the graduates of the schools in the smaller cities or towns? I would say to this, that the same system might be brought to bear in the small towns as in the large. In every community large enough to require and support a hospital for the sick poor, there must be a certain number of sick in wealthy homes requiring the services of the trained nurse. The mistake has been in leaving these towns and crowding into the large cities. So much so has this been the case, that our city registries are frequently called upon to send their nurses to the adjacent towns and smaller cities which might have been supplied by their own nurses trained and graduated within their own borders.

Now, after all is said and done, the truth remains that the greatest obstacle in the establishment of co-operative registries, or co-operative anything for women, is the woman herself.

Men in their work and struggle for life have learned through succeeding ages the lesson that union is strength, and that organisation means protection and success in their various trades and professions. They have learned to submit to rules and regulations when once they are formulated by general consent, and to bow to authority which they themselves have established They know how to cohere, to sustain each other; they congregate together for their common protection and safety.

Women have yet this great lesson to learn, and we, who as superintendents have gained some experience in management and organisation, owe it to our graduate nurses in this, their own special work of nursing, gives, so far as in us lies, and with all the influence our position gives, to teach them for their own safety and protection to co-operate and organise societies for mutual protection and advancement.

### The Royal National Pension Fund for Nurses.

### REPORT OF THE NINTH ANNUAL MEETING.

The ninth annual general meeting of the members of the Royal National Pension Fund for Nurses was held at the offices, 28, Finsbury Pavement, London, E.C., on Thursday, March 12th, 1896. Mr. Walter H. Burns, chairman of the council, presided, and there were, amongst others, present: Mr. Henry C. Burdett, Mr. Gerrard Norman, Mr. Arthur H. A. Morton, Mr. W. S. M. Burns, Dr. Herbert P. Hawkins, Mr. Norman, Mr. Edward Rawlings, the Hon. Herbert C. Gibbs, Miss Pritchard, and others.

The Secretary (Mr. Louis H. M. Dick) having read the notice convening the meeting, the report and balance-sheet, which had already been printed and circulated, were taken as read.

### The Chairman's Address.

Ladies and Gentlemen,—In commenting upon the report which has been laid before you, there is very little for me to add to it, as the year has been uneventful except in its continued record of unexampled prosperity.

The proposals received for pension show an increase of 305 over 1894, and the proposals received for sickness assurance an increase of 123. The premiums received amount to £11,000 more than during 1894.

The total investments, which were £202,000 at the end of 1894, amount to over £272,000 in 1895, and the expenses which reached £1,864 in 1894 have been reduced to £1,791 in 1895, showing that the working expenses are proportionately considerably less and well within the consulting actuary's loading. I am glad to say that during the year investments have been made of about £80,000 and about £10,000 of securities have been sold or paid off. The average return on the money invested during the year is a trifle over 4⅓ per cent. I need not say that to obtain this relatively high rate the greatest care and constant watchfulness have had to be exercised, and it is a matter of satisfaction to the council and myself to be able to inform you that, notwithstanding the many fluctuations and events of the past year, the securities are worth more than they cost us. Were our funds invested in Consols and other securities usually considered trustees' securities, the interest would be so low that we could not do nearly so well for the nurses as we have done, and while we have a very large discretion in our articles for the making of investments, I may say that the utmost care is exercised to vary them in as many ways as possible, and to endeavour to combine safety with a good return. So far the result of our care speaks for itself in the satisfactory outcome, and I trust, and see no reason to doubt, that it will continue to do so.

During the year we have had a great event in the reception of the third and fourth thousand nurses at Marlborough House. This undoubtedly accounts for a large proportion of the enormous increase in policies over 1894. The great kindness shown to the nurses by the Prince and Princess on these occasions is invaluable to the Fund in many ways. It leads many to join it. It also causes a very large paying up of premiums in arrear; and it gives us a publicity which is very valuable, as the amount which we spend on advertising is the merest trifle. Of course the great increase in premiums is also a natural consequence of a large number of new policies and of the reception at Marlborough House. I am sure I re-echo the feeling of all the nurses as well as of the council in again tendering to the Prince and Princess our hearty thanks for their unwearied kindness and protection, and I am glad to say that the good feeling of the nurses towards the Royal Family has taken a tangible shape in the presentation, by shilling subscriptions, of a very handsome tea-table to the Princess Maud upon her approaching marriage.

Of payments discontinued, there were 186, but of them seven were substituted policies; the number of nurses retiring or dying was 179. The causes were: Marriage, 29; giving up nursing, 54; deaths, 10; inability to keep up payments, 24; and various causes, 62. It is a pleasing feature to note that with scarcely an exception the letters from the nurses who have withdrawn testify to the great assistance which the Fund has been to them in saving money against the hour of need, and it is a satisfaction, in view of the fact, that it was expressly to provide this assistance to thrift that the Fund was instituted. I hope, however, that the nurses will not consider me indiscreet or intrusive in advising them most strongly not to withdraw their money from the Fund for the purpose of giving it to their relations. The temptation to do so is very great; but it is not for that purpose that the Fund has been started. It is to benefit them in their old age, and when a nurse under a kindly impulse takes out her savings and gives them away to people to whom they often do very little good, she deprives herself of the benefit of her self-denial, and it is not the province of the Fund to take all the trouble and all the risks on itself to benefit anyone but the nurses themselves.

During the year the sick pay has increased somewhat, amounting to £909, against £739, and we have been compelled to appoint a medical referee for cases, as we felt that the lay power of dealing with them was insufficient protection. We have appointed to the post Dr. Potter, and we feel confident that he will exercise due care, coupled with a kindly interest in the nurses.

The Benevolent Fund has been most efficient, and the devotion of the ladies in looking after it has been unceasing. Too much thanks cannot be given them by both the nurses and council. The demands upon the Fund are now very great, and it is absolutely necessary that during the year some steps should be taken to increase its amount. We have now 22 permanent pensioners upon the Fund, and we have distributed £399, against £425 last year—which is very nearly our entire income. The labour involved in receiving and inquiring into applications becomes greater year by year, and it will not be long before an increase of clerical assistance will be required for this department.

We have to deplore the death of Dr. Bristowe, to which allusion has been made in the official report. He had been a member of the council since the institution of the Fund, and no one has rendered it more service. We also regret the retirement of Mr. Charles Cotes owing to the pressure of other claims upon his time. To supply the vacancy created by the death of Dr. Bristowe, the council have elected Dr. Herbert P. Hawkins, of St. Thomas's Hospital. They have also strengthened the council by adding to it the Hon. Walter Rothschild, Mr. C. Eric Hambro, and Mr. W. S. M. Burns, sons of present members of the council, and who, we hope, will continue the traditions of management which their fathers will leave them.

Our Pension Fund investments alone now considerably exceed a quarter of a million. In cash we have returned over £20,000. In sick pay we have distributed more than £3,000. In pensions, already £2,363 has been paid.

The Benevolent Fund has given away over £1,500 to alleviate distress; and all this has been accomplished in the short space of barely eight years so far as the Pension Fund is concerned, and five years as regards the Benevolent Fund. There are visible indications on all sides of the rapidly increasing popularity of the Fund, and I have little doubt that during the coming year the new business will be far in excess of the average years prior to 1895. The Pension Fund is now firmly established, and may be considered a great national institution.

Mr. Morton: Mr. Chairman, ladies and gentlemen,—I have very great pleasure indeed in seconding the adoption of this report. I must say, for my own part, that I have always felt it a great honour and distinction to serve this noble institution. It would be a great satisfaction to me if we had had to-day several of the nurses present, such as could have been spared from the duties which they undertake so much for the benefit of society, in order that we might assure them of the present situation and state of this excellent society. There are only two points in connection with the report upon which I wish to say a word, both of which have been touched upon by our chairman in his speech. The first is the very important one with regard to the withdrawal of their funds by nurses. We have the best reason for knowing that those withdrawals have been very far indeed from satisfactory in their results. We know that in some cases those who have advised the withdrawals have promised an annual income of 5 per cent. upon the amount; but those who are acquainted with investments and the power of obtaining such a return as 5 per cent., know very well that such promises must be illusory, and had there been any nurses present here to-day, it would have been a great satisfaction to me to impress upon them the importance of deep consideration before ever withdrawing their funds from the care of the society. The other point that I should wish to dwell upon is one which the chairman, with his great knowledge and experience of such matters, is far more competent than I to speak about, and that is with regard to the investment of our funds. That is, indeed, of all others, the most important question which the council have to consider. I am connected with the London County Council, and a member of its Finance Committee. It so happened that yesterday we had to invest a very large sum of money on behalf of the Provident Fund connected with that institution; and it was impossible for us to obtain anything like the return which we have got here, and which, I am happy to say, from my knowledge of the investments here, is obtained only by the greatest possible care and with the greatest possible ability, and with, as I believe, the greatest possible safety. It is there that we shall have, probably, the greatest difficulty in the future, and where we ought to be grateful to have the support and the most valuable assistance of our chairman. I will conclude, gentlemen, by asking you to allow me to second the resolution.

The re-election of the retiring members of the council, Mr. H. C. Burdett, the Hon. H. C. Gibbs, Dr. H. P. Hawkins, Mr. E. M. Ind, Mr. A. H. A. Morton, and of Mr. F. Whinney (the auditor) was then proceeded with.

The Chairman: The ballot has taken place during the last two or three weeks.

The Secretary afterwards read the result of the ballot for annuitants and policy-holders' represesentatives. He said 1,625 voting cards were returned, of which 27 were unsigned, 1,222 were invalid because they contained eight or more names, and 376 were in order.

The voting gives the following results: Miss L. M. Gordon received 328 votes, Miss K. H. Monk 303, Miss E. Vincent 281, Miss E. Fisher 273, Miss C. Davidson 272, Miss F. C. Nott-Bower 263, and Miss M. Cave 245. Other ladies to the number of sixteen also received votes; in one case 233 votes, in another seven, in two cases five, in one case two, and in eleven cases one vote each.

Mr. H. C. Burdett: Mr. Chairman, I notice that no representative of Ireland has been included. The lady who was proposed came out eighth. Now, there are eight vacancies, and as we have a number of representatives in Ireland, I think in the exercise of the authority we have we should fill up the eighth vacancy by adding Miss Dunn's name. I do not know whether you see any objection to that. There are eight vacancies, and we have only filled up seven.

The Chairman: But in those eight is not one to be named by the council, and seven by the representatives themselves?

The Secretary: The names of those elected last March were mentioned, and also the eighth name was given as the nurses' nominee. There are no names suggested by the council at all.

The Chairman: Then why did this lady fail to be elected?

The Secretary: She did not get sufficient votes.

Mr. Burdett: But we have power to elect an eighth here. Ireland is at present excluded altogether, and I think that is very undesirable.

The Chairman: We sent out for election eight names, and seven have been elected.

Mr. Burdett: Yes, but there are eight vacancies under our articles. The only way, I think, in which we can deal with Miss Dunn is to say that seven papers have been sent out, but that an eighth vacancy has occurred, which we will now fill.

The Chairman: First of all, I think we should elect the seven. I should propose that we declare these seven ladies elected, and deal with the eighth name later on.

Mr. Gibbs: If it is for the council to deal with the matter, I think the best way would be to leave it to be dealt with at the next council meeting.

The Chairman: I do not know whether it is to be dealt with by the council or by the general meeting.

Mr. Burdett: We have only had seven elected so far, and the articles provide: "That from and after January 1st, 1890, there shall be added to the council not more than eight other persons, which persons are hereinafter referred to as the representatives of annuitants and policy-holders in this society." Therefore, I take it, we have power at this meeting to elect an eighth representative.

Mr. Norman: Then what was the object of putting in the provision that if a member voted for more than seven the vote should be void?

Mr. Burdett: Because there are only seven vacancies unless the meeting of members decide otherwise. There were seven, and I want to add one more.

Mr. Gibbs: Would the meeting be prepared to leave it to the discretion of the council?

The Chairman: The council, I understand, has no power.

Mr. Gibbs: But we could authorise the council, if they thought fit to do so, to elect an eighth representative.

The Chairman: How many votes did Miss Dunn receive?

The Secretary: 233.

The Chairman: Then practically she proved herself to be satisfactory to the annuitants?

The Secretary: Yes; she was only twelve votes under the seventh nominee.

The Chairman: I think we ought simply at present to put these seven names to the vote, or, rather, to declare them elected. That we have power to do; but the question of electing an eighth representative is a distinct and separate business. I think it rests with the meeting to appoint an eighth; but I would point out that this meeting is composed almost entirely of members of the council, and, therefore, it cannot be properly said to be a representative meeting of annuitants. Perhaps we might defer it for another year before increasing the number to eight. I imagine, as Mr. Burdett referred to her as an Irish representative, Miss Dunn would very seldom be here?

Mr. Burdett: Oh, yes; she is a regular attendant, and one of our most active members; and I may say that since she has been elected one of the most important organisations of nurses in Ireland has become affiliated with this Fund. Of course, we have not at present so many Irish as English members, and that accounts for her having missed election by a few votes. I think, on the whole, it would be best to accept these seven, and empower the council to elect an eighth at their discretion. Mr. Nairne can advise us upon it, but it clearly must be done at a meeting of members.

The Chairman: It would be simpler to send out another card with this lady's name, and ask the annuitants to vote upon it.

Mr. Burdett: I should not think it would be necessary to go to that expense. I beg to move, sir, "That the council be empowered, at their discretion, and on the advice of the solicitor, to elect an eighth member, to be added to the seven selected as the nurses' representatives."

Mr. Nairne: I would suggest that it ought to be "to elect or to take steps to elect," because in my judgment, at present, under this Article 29, the election should be by the annuitants and policy-holders. There is nothing that I see to prevent your having another election to fill up the eighth place.

Mr. Gibbs: I think Mr. Burdett means that the whole thing should be left in the discretion of the council.

Mr. Burdett: Certainly.

Mr. Gibbs: Then I beg to second that resolution.

The Chairman: I do not think the council has power. If you leave it subject to the decision of the solicitor it can do no harm. As at present advised, Mr. Nairne thinks we are not authorised to do it. That was the very object of creating that body of representatives, that they should be distinct from the council.

Mr. Nairne: Quite so.

The Chairman: While a resolution is being drafted to carry that out we may go on to the next business, which is to pass a vote of thanks to Mr. Dick, the secretary, and the staff, and also to the ladies and the secretary of the Benevolent Fund. It is impossible for me to convey to you the devotion and attention to business which all these friends have given to us. When I tell you that the correspondence and detail in this office is greater even than in a bank, that something like 75,000 to 100,000 letters pass through this office every year, and the entries are innumerable in the books, as you can readily imagine from the fact that we receive small premiums on over 4,000 policies—with the small staff we have, I do not think it is possible to exaggerate the satisfactory labour which the secretary and his assistants have given. As to the Nurses' Benevolent Fund, which has been entirely looked after by the ladies who have taken charge of it under Lady Rothschild, I have never heard a single complaint from the nurses, and I am personally aware of how regular is their attendance at the meetings, and how carefully they examine the cases brought before them. I therefore think it is only the duty of this meeting to record their thanks to these friends and helpers in the cause. I beg to move to them a vote of thanks.

Mr. Norman: I have great pleasure in seconding that.

The resolution was unanimously carried.

The Chairman: I have now the resolution which has been prepared as to the election of an eighth representative. Mr. Burdett moves, and Mr. Gibbs seconds, "That, subject to the solicitor's opinion, the council be authorised to take steps to secure the election of an eighth representative of the annuitants and policy-holders in this society."

The resolution was duly carried.

Mr. Burdett: Ladies and Gentlemen,—I have much pleasure in proposing a vote of thanks to Mr. Walter H. Burns, our chairman. Those of us who are members of the council know how much we owe to him and to his family, so far as this Fund is concerned. Mr. Burns is untiring in his devotion to the interests of the Fund. In giving his great experience and his valuable time to carefully looking after the whole of our investments, he does us a service the value of which it is impossible to over-estimate. (Cheers.) I feel very keenly that no words could accurately describe our indebtedness to Mr. Burns. I am quite sure that it has been to me a matter of the sincerest congratulation that at the request of the late Mr. Julius S. Morgan—I remember the incident well—Mr. Burns, after some hesitation, kindly consented to undertake the responsible duties of chairman of this Fund; and I venture to think that neither he nor any of us realised at that time how much work and how much earnest endeavour would be entailed upon him by his undertaking that position. Certainly, the greater the responsibility, and the more time that has been required, the greater has been the willingness with which it has been tendered. And I do feel and say, on behalf of the nurses as well as on behalf of ourselves, that we owe to Mr. Burns a most deep and cordial feeling of gratitude for his really inestimable services. I, therefore, have very great pleasure—in no formal way, but in the heartiest manner—in proposing a vote of thanks to the chairman of the day, Mr. W. H. Burns, who is also the chairman of our council.

Dr. Hawkins: I sir have the greatest pleasure in seconding this vote of thanks to you. I am not familiar with the course of business of councils of this kind, but I must say that, as a junior member, I cannot conceive of more business-like work than the council has done under your chairmanship.

The resolution was carried with acclamation.

The Chairman: I am extremely obliged to Mr. Burdett and Dr. Hawkins for the very kind way in which they have spoken about my very feeble services. I have given my work to the cause partly out of respect for the memory of Mr. Morgan, whom I greatly admired, partly out of personal friendship for Mr. Burdett, and those who urged me to take this position; but, since I have joined the council, I have given it for love of the cause itself. I have seen that it is a good cause; that it is not simply a device for extracting more or less money from the pockets of the rich to be handed over in a charitable way to people who do not deserve it, and who are pauperised by it; but it is an aid to people who are equally struggling with ourselves to raise and better their position, and to gather money together for their old age. It is not in any sense a charity. It is only the assistance of those who are richer and better placed from their business experience to give their advice to people who are working as faithfully as we are ourselves, and in causes which perhaps do more good to the community than we ourselves are doing. While I have strength, and you are willing to have my help and work and guidance, I promise you my very best assistance in the future as I have endeavoured to give it in the past. (Applause.)

The proceedings terminated.

### Novelties for Nurses.

### AN OPPORTUNITY FOR NURSES.

Shoes and boots are very expensive items in nurses' apparel. It is false economy to buy inferior articles at any price, but occasionally opportunity offers of making good purchases when a clearance sale takes place at a first-class establishment. We are informed that the London Shoe Company, at 116, Bond Street, is disposing of a surplus stock on March 23rd and 24th, when some hundreds of pairs of boots and shoes are to be sold at half price.

### Our American Letter.

During the past month the old St. Luke's Hospital, New York, of which the corner-stone was laid in 1854 by Bishop Wainwright, has been definitely closed, and the patients transferred to the splendid new building, equipped with every modern appliance, at 113th Street and Amsterdam Avenue, which will for the future represent that institution. This model hospital would have delighted the heart of Dr. Muhlenberg, whose warm interest and untiring exertions resulted in the founding of the original St. Luke's.

The most interesting event recently in nursing circles here has been the third annual convention of the Society of the American Superintendents of Training Schools, held at Philadelphia, Pa. Very able papers were read, some of which readers of The Hospital will have been able to see in full for themselves, and interesting discussions on the various subjects took place amongst the fifty superintendents who were present at the meetings.

The annual meeting of the Alumnæ Association of the New York City Training School was held in February, when the question as to provision for sick members was fully discussed, an interesting report being given of the system followed by some of the principal nurses' alumnæ in America. The association has lately had to mourn the deaths of two valued members—Mrs. Mary Dowling Lester, one of the earliest members and on the board of trustees, and Miss Rose Shearer.

The Advisory Board of the Training School for Nurses in connection with the Orange Memorial Hospital are proposing to erect a special building as an infirmary for the use of the graduate and undergraduate nurses of the school, if a sufficient sum of money can be raised for the purpose. The proposition originally came from the Guild of St. Barnabas for Nurses, but for obvious reasons it has been decided that such an infirmary will be best provided by the authorities of the school itself rather than by another society.

The progress of Miss Clara Barton's mission to the Armenians has been watched with considerable interest by her friends in the States. Upon her arrival in Constantinople Miss Barton had an interview with the Turkish Minister of Foreign Affairs, and was assured that the Porte would not place any difficulties in the way of the distribution of relief to the needy in Armenia, after which she proceeded with her journey into the interior.

The annual meeting of the Bellevue Training School Alumnæ Association, held at the Nurses' Home, East Twenty-sixth Street, was a very successful affair, and many friends attended the reception held by Miss Brennan.

An important order has lately been made by the New York State Commissioners of Lunacy by which the employés of State hospitals are required to pass an examination, all the papers being dealt with at headquarters. Successful candidates will be called "nurses," while those who do not pass will be "attendants," and to the latter will belong duties connected with the superintendence of meals and dining-rooms. They will have nothing to do with the care of the patients.

Michigan is in the very van of sanitary progress, mainly, it must be said, owing to the energy of a woman, Dr. Mary E. Green, and a law has been enacted for the compulsory teaching in all schools of the State the "modes by which the dangerous communicable diseases are spread, and the best methods for the restriction and prevention of such diseases." Throughout the whole State the keen interest taken by women's clubs and societies in sanitary reform is a peculiar characteristic, and the "campaign of education" carried on by the Board of Health numbers women among its most ardent supporters.

### Royal British Nurses' Association.

The fourth sessional lecture of the season will be delivered on Friday, March 20th, at eight p.m., at the offices of the association, 17, Old Cavendish Street, W., by Miss De Pledge, matron of the Chelsea Infirmary, the subject being "The Village of Palaces." Members are admitted free, the general public on payment of one shilling.

### East End Mothers' Home.

ANNUAL MEETING.

By the kind permission of the Hon. W. F. D. Smith, M.P., the annual meeting of the East End Mothers' Home, Commercial Road, Stepney, was held on March 17th, at 3, Grosvenor Place. There was a good attendance, the chair being taken by Lord Medway, who spoke of the charity as one which could do nothing but good, and did not in any way pauperise those who benefited by it. Besides the in-patients so successfully nursed in the home, many poor mothers were attended by the district midwives in their own homes. By this means it was possible to convey some knowledge of sanitary precautions to the patients and their friends. The new premises recently added to the East End Mothers' Home were most satisfactory, but they had, of course, added greatly to the year's expenses.

The Secretary presented the report and audited balance-sheet, mentioning that only nine maternal deaths had occurred in eleven years.

The adoption of the report was proposed by Dr. Champneys, who said that he had inspected the home with satisfaction, and considered that the training of pupil midwives was, perhaps, the most valuable part of the work done in connection with it. The excellence of the training was shown by the fact that all the pupils sent up for the London Obstetrical Society's examination had passed, and as this was a thorough and efficient examination it showed that the candidates had received good instruction. He considered the work done in the homes of the poor was excellent, and introduced sanitary and antiseptic precautions as well as general decency. The low death rate was most satisfactory, as those persons admitted to the home might be described as "picked bad cases."

In seconding the adoption of the report the Rev. F. J. Jomini spoke of the great difficulties of East-end work, for owing to the high rents the people were unable to have proper accommodation and were much crowded together. The Mothers' Home was, therefore, a great boon to the women, who secured quiet and good care there in the hour of need. The infants were also greatly benefited, and so, indirectly, were the fathers, who gladly acknowledged the blessing of such a place and the very great kindness exhibited to their wives. He considered this a great tribute, as the poor were not always the easiest people to please. The report was adopted unanimously.

The re-election of committee and honorary officers was proposed by Canon Eyton, seconded by Mr. John Dickinson, magistrate of the Thames Police Court, and carried.

A vote of thanks, proposed by the Rev. Marmaduke Hare, to the chairman for presiding, and to the Hon. W. F. D. Smith, M.P., for the loan of the room, was seconded by Mr. Percy Wigram, and carried.

### Practical Points.

"*Perplexed One*" *writes*: *Will you give me your advice in the following circumstances? A trained nurse, with several years' experience in district work, is engaged by a committee to work in a village. She discovers in taking up her duties that the one medical man is not (for private reasons) in favour of the scheme, and neither he nor his wife are on the committee, the work being carried on by a "Lay" Committee, headed by the Vicar, who wishes to help the sick poor in his parish. Should the nurse resign, she will receive no testimonial from the medical man. Would that prevent her from obtaining another post, and ought she, under the circumstances, to resign?*

It cannot be held that the fact of the one medical man in a village disapproving of district nurses should debar the villagers from the advantages of good nursing. But such passive opposition makes the position of a nurse very trying, and one requiring the greatest tact. Probably the only point on which such a nurse need insist is that her services should never be given unless asked for by the people in the house where the patient dwells. If the nurse allows herself to be sent to a doctor's patients by a committee when not desired by the patients themselves there will be no peace; but if she only goes where the people themselves ask for her services, and if she then behaves with tact and judgment, the chances are that in six months she and the doctor will be good friends.

### Everybody's Opinion.

[Correspondence on all subjects is invited, but we cannot in any way be responsible for the opinions expressed by our correspondents. No communications can be entertained if the name and address of the correspondent is not given, or unless one side of the paper only be written on.]

"THE NURSES' JOURNAL."

"A Member of the Council" writes: Will you allow me to express through your paper my great surprise at not seeing an account of the special meeting of the R.B.N.A., summoned by H.R.H. the President, on January 28th last, to consider the "Barlow case," in the current issue of *The Nurses' Journal?* I have always understood the above journal was one belonging to the Association, giving accounts of its meetings, &c. Therefore I cannot understand the omission of such an important meeting.

[*** We have made inquiries, and are informed that the omission in question arose as follows: On February 14th the Secretary to the R.B.N.A. received a communication from Messrs. Mear and Fowler, solicitors for Miss Barlow, announcing their intention of applying for an injunction to restrain the Association from publishing in *The Nnrses' Journal* an account of the special meeting summoned by H.R.H. the President on January 28th last, unless an undertaking was given to the contrary. This communication reached the secretary after *The Nurses' Journal*, which had been unavoidably delayed, would, in the ordinary way, have gone to press. The resolution which it was alleged was libellous had already appeared in The Hospital and *British Medical Journal*, with an account of the proceedings at the meeting in question, against neither of which journals had any legal proceedings been taken. An opinion, however, prevailed that it would be advisable to withhold the publication in *The Nurses' Journal* until legal opinion had been obtained, and so it was omitted from the February number. We regard this non-publication as unfortunate, because a fair account of the proceedings at the January meeting in the February number would have been privileged, but a three months' delay must cause the matter to cease to be of immediate public interest, and to print it in the May number of *The Nurses' Journal* may render the proprietors liable to damages under the Newspaper Libel Acts. It is surely time that the R.B.N.A. made arrangements to appoint or to secure the services of a capable solicitor who will be available to give immediate and prompt assistance on any similar occasion in future.—Ed *T. H.*]

### District Nursing in Ireland.

The following graphic description from a Donegal District Nurse of "the perils in the sea" which befel her in the course of her daily work is published in the recently-issued annual report of St. Patrick's Home, Dublin:—

I have been very busy on Arranmore with three bad cases. . . . I must tell you that we (two Arran men and myself) were very nearly lost yesterday evening coming home. A most terrible hurricane arose, and we were just off Arran in the midst of rocks and breakers. It came cn suddenly, and the sea rose ever so quickly; we just kept her head to wind, and trusted to God, for we were powerless. A good big fishing-boat she was, but the waves were breaking all around, and we could hardly see, with a dark fog of rain pouring on us. I had my cloak off, for fear it would be in my way of steering. Finally, when the rain stopped, and we could see, we let her drift, the two men trying hard to hold her back with oars, and I steering, till we got to back of Rutland and safety. The men then pulled for a mile, till we got to a place we could land.

Words would fail me to describe the two hours we were out, tossing about in a raging sea. The men were very good, and tried to cheer me by saying, "Don't be afraid, nurse; we will get ashore yet," when I knew they had lost all hope. However, none of us lost our heads, and we are all right today with the exception of a few bruises, and feeling shaky.

### Appointments.

Mill Road Infirmary, Liverpool.—Mrs. Price, late Sister Clinical, Guy's Hospital, has been appointed Matron of this infirmary.

Royal West of England Sanatorium, Weston-super-Mare.—Miss Maine, whose appointment to the post of Lady Superintendent of this institution has been already announced in this column, asks us to state that her Christian name is Edith, not Ethel, as erroneously given last week.

### Minor Appointments.

Dreadnought Hospital, Greenwich.—Miss Florence Worley has been appointed Sister of the accident floor and theatre. Miss Worley was trained at St. Bartholomew's Hospital, subsequently holding there the position of staff nurse, and has since been charge-nurse at the Swanley Convalescent Home.

Chichester Workhouse.—Nurse Elizabeth M. Renahan has been appointed Staff Nurse at this workhouse. She was trained at Queen's Hospital, Birmingham, and has since worked at the Kent and Canterbury Hospital, at the City Infirmary, Birmingham, and at the Mill Road Infirmary, Liverpool.

Northern Hospital, Winchmore Hill.—Miss Mary Leigh Swift has been appointed Night Sister at this hospital. Miss Swift received her training at Guy's Hospital, afterwards holding the post of charge nurse at the Liverpool City Fever Hospital, the Mill Road Infirmary, Liverpool, and the Northern Hospital, Winchmore Hill. We cordially congratulate Miss Swift on her promotion.

### Notes and Queries.

### Queries.

(189) *Nauheim Treatment.*—(1) Can you give me the address of a home where the Nauheim treatment is given? (2) Will you also advise me as to obtaining work? I am a fully-trained nurse and have been taking private cases on my own account, but find the work very uncertain.—*Troublesome.*

(190) *Holidays for Nurses.*—I am preparing to receive nurses for change and rest in my house in Surrey. Will you tell me if nurses often require such a home, and how I can make the fact known to those who do?—*Midwife, L.O.S.*

(191) *Training.*—Can you tell me of any hospital where probationers are received at the age of 20, and will you also tell me if all nurses are required to be above a certain height?—*E. M. H.*

(192) *Ward Prayers.*—Can you tell me of a short and suitable form of service convenient for daily use at morning prayers in a home for incurables?—*Secretary.*

(193) *India.*—Will you tell me the best way for two nurses to work their way out to India, and the best Indian papers to advertise in? We are thoroughly trained in medical, surgical, and midwifery work, and are strong and healthy. Could you also tell us what fees we might expect to get working in India on our own account?—*R. N. P. F.*

(194) *Training.*—I want to get into a children's hospital; where can I apply? I have bought "How to Become a Nurse," but that does not give many children's hospitals. Are probationers required to have any knowledge of Latin and Greek?—*A. S.*

### Answers.

(189) *Nauheim Treatment* (*Troublesome*).—(1) Write to Dr. Bezley Thorne or Dr. Fletcher Little, whose addresses you will find in the "Medical Directory." See also advertisement under "Residential Homes" in the Nursing Mirror for March 14th. (2) Private nursing is often very precarious, and unless you have definite promise of work it would be very unwise to come to London. Why not try for a post as staff nurse in a provincial hospital or poor law infirmary? Watch the columns in The Hospital. There are many advertisements every week which you might answer.

(190) *Holidays for Nurses* (*Midwife, L.O.S.*)—Advertise in our columns and make the advantages you offer to nurses known as widely as possible among friends. We should think many nurses would be glad of such an opportunity.

(191) *Training* (*E. M. H.*)—We shall be happy to answer this query on receipt of the full name and address of the writer.

(192) *Ward Prayers* (*Secretary*).—Write to Masters and Co., 78, New Bond Street, for catalogue. There is a "Hospital Service Book" which you can obtain from them which would, we fancy, meet your requirements.

(193) *India* (*R. N. P. F.*)—You will be be very ill-advised if you go out to India on the chance of private nursing without any certain and definite prospect of work when you get there. Such a venture is almost certain to end in failure. Get "How to Become a Nurse" (Scientific Press, 428, Strand, W.C.) and read the chapter on "Nursing Abroad," and you might write to the Hon. Mrs. Lyttleton, secretary to the Up-Country European Nursing Association, 21, Carlton House Terrace, for particulars. That association was started with the intention of supplying trained nurses for private work in India.

(194) *Training* (*A. S.*).—If you have applied unsuccessfully for admission into all the twenty-five children's hospitals given in "How to Become a Nurse," in London and the provinces, the fault must surely lie in yourself. What is your age? And of what do you think a nurse's duties consist that a knowledge of Latin or Greek should be demanded of her? Read Miss Lückes's "Lectures on Nursing."

### For Reading to the Sick.

### FAITH.

The cry of "God wills it" must be the eternal watchword of every undertaking."—*Mazzini.*

**Verses.**

Thro' silence and the trembling stars
Comes Faith from tracts no feet have trod.
—*Tennyson.*

Simple rule, and safest guiding,
Inward peace, and inward light;
Star upon our path abiding,
Trust in God, and do the Right.
—*Norman Macleod*

Look full into thy spirit's self,
The world of mystery scan;
What if thy way to Faith in God
Should lie through faith in man! —*Bright.*

Be bounteous in the Faith, for not mis-spent
Is confidence unto the Father lent;
Thy need is sown and rooted for His rain . . .
Work on! One day, beyond all thought of praise,
A sunny joy will crown thee with its rays;
Nor other than thy need, thy recompense.
—*Macdonald.*

Self is earthly, Faith alone
Makes an unseen World our own;
Faith relinquished—how we roam,
Feel our way, and leave our home!
Spurious gems our hopes entice,
While we scorn the pearl of price;
And preferring servant's pay,
Cast the children's bread away. —*Cowper.*

Therefore love, and believe; for works will follow spontaneous,
Even as day does the sun. The right from the good is an offspring—
Love in a bodily shape; and Christian works are no more than
Animate love and faith, as flowers are the animate springtide.
—*Longfellow.*

**Reading.**

It is a noble act of faith to say "I shall not want." God honours that confidence which honours Him; He answers it with His blessing.—*Rev. J. Stevenson.*

Many people never learn to see much with their natural eyes. They walk over the fields in summer days, and never see a lovely thing; while in every wild flower and in every grass blade there is beauty enough, if perceived, to fill the dullest heart with rapture. It is still more true in spiritual things; we walk in a world full of the glories of God's love; yet how much do we see of this ineffable splendour? At best, in this world we see things only through a glass, darkly. Should we not train our eyes to see?—*H. J. Miller, D.D.*

In all deep grief this is the truest way to find comfort—"Just to cling to Christ in the darkness and believe." There is no need to ask questions, for no one can answer them. There is no use to strain our eyes trying to see the light, for as yet there is no light to see. All we can do is just to throw ourselves on our Saviour's bosom, and lie there till the light breaks. We can always be sure of the love and the faithfulness of Christ. We may nestle down, as John did, upon the Saviour's bosom, and be quiet and confident in the time of our sorest calamities. "In the world ye shall have tribulations. In Me ye shall have peace."—*H. J. Miller, D.D.*

Let us take to ourselves. Wherever faith in Christ is, there is Christ Himself. He said to Martha, "Believest thou this?" Wherever there is a heart to answer, "Lord, I believe," there Christ is present. There our Lord vouchsafes to stand, though unseen—blessed be His name! Nothing can rob us of this consolation: we will be as certain, through His grace, that He is standing over us, in love, as though we saw Him. We will not doubt an instant that He is thoughtful about us.—*J. H. Newman.*

## Figures and Tables

**Figure f1:**